# LDL-cholesterol change and goal attainment following statin intensity titration among Asians in primary care: a retrospective cohort study

**DOI:** 10.1186/s12944-020-01427-z

**Published:** 2021-01-06

**Authors:** Hao Sen Andrew Fang, Qiao Gao, Mong Li Lee, Wynne Hsu, Ngiap Chuan Tan

**Affiliations:** 1 SingHealth Polyclinics, SingHealth, 167, Jalan Bukit Merah, Connection One, Tower 5, #15-10, Singapore, P.O. 150167 Singapore; 2 School of Computing, National University of Singapore, Singapore, Singapore; 3 Institute of Data Science, National University of Singapore, Singapore, Singapore; 4 Family Medicine Academic Clinical Programme, SingHealth-Duke NUS Academic Medical Centre, Singapore, Singapore

**Keywords:** LDL-cholesterol, Statin, Percentage change, Asian, Real-world data, Goal attainment, Primary care

## Abstract

**Background:**

Clinical trials have demonstrated that either initiating or up-titrating a statin dose substantially reduce Low-Density Lipoprotein-Cholesterol (LDL-C) levels. However, statin adherence in actual practice tends to be suboptimal, leading to diminished effectiveness. This study aims to use real-world data to determine the effect on LDL-C levels and LDL-C goal attainment rates, when selected statins are titrated in Asian patients.

**Methods:**

A retrospective cohort study over a 5-year period, from April 2014 to March 2019 was conducted on a cohort of multi-ethnic adult Asian patients with clinical diagnosis of Dyslipidaemia in a primary care clinic in Singapore. The statins were classified into low-intensity (LI), moderate-intensity (MI) and high-intensity (HI) groups according to the 2018 American College of Cardiology and American Heart Association (ACC/AHA) Blood Cholesterol Guidelines. Patients were grouped into “No statin”, “Non-titrators” and “Titrators” cohorts based on prescribing patterns. For the “Titrators” cohort, the mean percentage change in LDL-C and absolute change in LDL-C goal attainment rates were computed for each permutation of statin intensity titration.

**Results:**

Among the cohort of 11,499 patients, with a total of 266,762 visits, there were 1962 pairs of LDL-C values associated with a statin titration. Initiation of LI, MI and HI statin resulted in a lowering of LDL-C by 21.6% (95%CI = 18.9–24.3%), 28.9% (95%CI = 25.0–32.7%) and 25.2% (95%CI = 12.8–37.7%) respectively. These were comparatively lower than results from clinical trials (30 to 63%). The change of LDL-C levels due to up-titration, down-titration, and discontinuation were − 12.4% to − 28.9%, + 13.2% to + 24.6%, and + 18.1% to + 32.1% respectively. The improvement in LDL-C goal attainment ranged from 26.5% to 47.1% when statin intensity was up-titrated.

**Conclusion:**

In this study based on real-world data of Asian patients in primary care, it was shown that although statin titration substantially affected LDL-C levels and LDL-C goal attainment rates, the magnitude was lower than results reported from clinical trials. These results should be taken into consideration and provide further insight to clinicians when making statin adjustment recommendations in order to achieve LDL-C targets in clinical practice, particularly for Asian populations.

## Background

The association between lowering of Low-Density Lipoprotein-Cholesterol (LDL-C) and reduction of cardiovascular diseases (CVD) is well-established [1, 2]. Clinical practice guidelines on Dyslipidaemia generally advocate lowering LDL-C to treatment goals based on each individual’s CVD risk [3–6]. The recommended LDL-C treatment goals, according to the 2019 European Society of Cardiology (ESC)/ European Atherosclerosis Society (EAS) Guidelines are < 1.8 mmol/L and < 1.4 mmol/L for patients with high and very high CVD risks respectively [4].

Statins are the first-choice medication for Dyslipidaemia [7–9]. Statin therapy is divided into three intensity levels: low-intensity (LI), moderate-intensity (MI) and high-intensity (HI), depending on the statin type and dosage [3]. In clinical trials, statin initiation has been shown to effectively reduce LDL-C levels by between 30 to 63%, while the doubling of dose further decreases it by 6% [10–15]. Given that these trials enrolled subject based on stringent eligibility criteria and reported on predominantly Caucasian populations, it remains uncertain if the magnitude of LDL-C lowering differ in actual clinical practice due to suboptimal medication adherence, variability in patient demographic characteristics, psychosocial profiles and health-seeking behaviour [16, 17]. A retrospective cohort study by Toth et al. involving an American managed-care population of largely Caucasian patients with high CVD risks on statin therapy reported that patients who up-titrated their statins had an average reduction of LDL-C levels by 9.6% [18]. No further analysis was conducted on LDL-C reduction for up-titration of different formulations of statin across different intensity levels.

Asians have different vascular risk profiles from Caucasians [19, 20]. South Asians, including Indians, have an excess risk for coronary artery disease beyond the currently known risk factors [21, 22]. It becomes critical to understand the effectiveness of statin in managing the Dyslipidaemia in Asians. In addition, statin adherence by patients tends to be suboptimal in the real world due to multiple reasons [23]. Kang et al. revealed that 45.3% of Asian patients reported poor adherence to their medications in a public primary care clinic in Singapore [24]. Another primary care study showed that 27.6% of the Asian study population failed to achieve LDL-C treatment goals despite on statins [25, 26]. Poor adherence to statins diminishes its effectiveness on the management of dyslipidaemia in routine clinical practices.

## Methods

### Study aim

This study aims to determine the magnitude of LDL-C change and effect on LDL-C goal attainment following titration of statin doses across the different intensity level among Asians who were managed in primary care for Dyslipidaemia.

### Study design, setting and population

A retrospective cohort study was conducted using patient electronic medical records (EMR) from a typical polyclinic located in south-eastern Singapore. This polyclinic manages about 450 to 500 patient attendances daily during office hours and serve about 350,000 multi-ethnic Asians (76.2% Chinese, 15.0% Malays, 7.4% Indians, 1.4% minority ethnic groups) living in the adjacent estates. About one-third of patients who attended the polyclinic are aged 65 years and above.

Based on local clinical practice guidelines, patients with Dyslipidaemia are reviewed by the polyclinic physicians and nurse clinicians every 3 to 6 months and are recommended to undertake laboratory investigations to assess their lipid profiles either once or twice annually, with flexibility for closer monitoring if their medical conditions are unstable [6]. Their demographic, clinical and laboratory information are collated and documented in the polyclinic EMR system.

The study population comprises multi-ethnic adult patients, aged 21 years or older, with clinical diagnosis of “Hyperlipidemia, unspecified” (International Classification of Diseases, 10th Revision [ICD-10] code: E78.5) documented in the EMR. Their clinical data from April 1, 2014 to March 31, 2019 at the study site were extracted from the EMR. Patients who had taken lipid lowering medications apart from statin, such as fibrates, ezetimibe, PCSK9 inhibitors, niacin and omega-3 fatty acid ethyl esters were excluded.

### Data definition

The statin intensity levels were classified based on statin types and dosage (Table 1), with reference to the 2018 American Heart Association / American College of Cardiology (AHA/ACC) guidelines [3]. The exceptions were extreme high doses of simvastatin which was categorised as HI, and extreme low doses of atorvastatin and rosuvastatin which were categorised as LI. As some patients were prescribed variable statin doses across different days of the week, the mean daily dose over a week was used to derive the statin dose. Additionally, a patient was considered to have discontinued taking statin if there was no repeat statin prescription within a year of the last prescription.

**Table 1:** Statin intensity level groupings based on types and doses of statins

Type of statin (x = dose in mg)	Low-intensity (LI)	Moderate-intensity (MI)	High-intensity (HI)
Pravastatin	0 < x ≤ 40	x > 40	NA
Lovastatin	0 < x ≤ 40	x > 40	NA
Simvastatin	0 < x ≤ 20	20 < x ≤ 80	x > 80
Atorvastatin	0 < x ≤ 10	10 < x < 40	x ≥ 40
Rosuvastatin	0 < x ≤ 5	5 < x < 20	x ≥ 20

*LI* Low-intensity statin, *MI* Moderate-intensity statin, *HI* High-intensity statin, *x* Statin dose in milligrams.

Patients were classified into CVD risk groups – low (LR), medium (MR), high (HR) and very high (VHR) – using a modified Framingham risk calculator. This modification had been carried out by local health authorities and has been adopted into local practice guidelines [6].

### Data processing

The aim of this study was to investigate the effect of statin intensity titration on the lowering of patient LDL-C. Patients with fewer than two LDL-C values were excluded as at least two LDL-C values were needed for comparison of statin effect. Patients who were not on statin therapy throughout the study period were grouped into the “No statin” cohort. For the remaining patients, statin intensity titration was determined by analysing their statin prescription records. The records were sorted by patient identifier and then by prescription date. Next, the time interval between two consecutive prescriptions for each patient were assessed for statin discontinuation. A discontinuation was defined as a gap in statin prescription of more than 1 year. Considering only consecutive prescriptions which were not labelled as discontinuation, a check for difference in statin intensity between prescriptions in each pair was then performed to identify statin intensity titrations. Patients with without any titration identified over the sampling period were grouped into the “Non-titrators” cohort, while the rest were grouped into the “Titrators” cohort.

For each patient in the “Titrators” cohort, all occurrences of statin intensity titration were first identified. For each statin intensity titration, a pair of LDL-C values *(pre-LDL, post-LDL)* was identified. *Pre-LDL* was defined as the most recent LDL-C result within 1 year before a statin intensity titration, and *post-LDL* was defined as the first LDL-C value within 6 weeks to 1 year after the statin intensity titration. The LDL-C values measured within 6 weeks of titration of a statin therapy were excluded. This 6-week window was chosen because clinical trials for lipid-lowering agents typically have a minimum 6-week follow-up to assess LDL-C lowering efficacy [18]. The 1 year restriction was used as a criterion in concordance with local guidelines’ recommendation to perform lipid panel tests annually for patients with Dyslipidaemia [6].

**Fig. 1:**
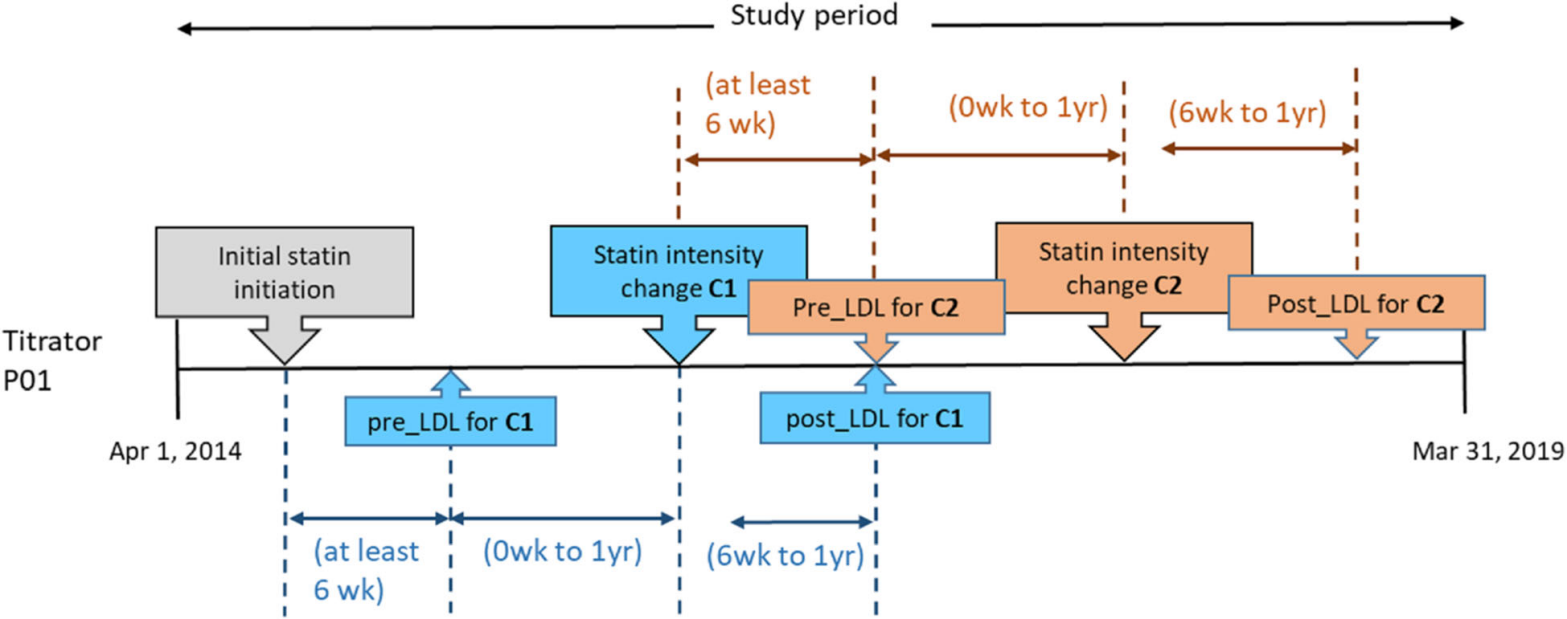
This figure illustrates the data processing to identify LDL-C pairs for patients in “Titrators” group. To obtain the LDL-C value before statin intensity change (pre_LDL) and after statin change (post_LDL) for the analysis, a statin intensity change is first identified. In the figure, two statin intensity changes (C1 and C2) were identified for the patient (P01). For each statin intensity change, the pre_LDL is taken to be the most recent LDL-C result within one year before a statin intensity titration, while the post_LDL is the first LDL-C value within six weeks to one year after the statin intensity titration. Abbreviations: *C1* first statin intensity change, *C2* second statin intensity change, *P01* illustrative patient, *pre_LDL* LDL-C before statin intensity titration, *post_LDL* LDL-C after the statin intensity titration

Since patients could have multiple statin intensity titrations, an LDL-C value could serve both as the *post-LDL* for one titration, as well as the *pre-LDL* for a subsequent titration. Fig. 1 illustrates two statin intensity titrations – C1 and C2 – and how the LDL-C pairs for the titrations are identified.

Although patients in the “Non-titrators” and “No statin” cohorts did not have any statin intensity titration, their LDL-C values could still trend over time. They were hence used as a comparison group. Similar to the “Titrators” cohort, pairs of consecutive LDL-C values were used to create the *(pre-LDL, post-LDL*) pair, with a minimum 6-week window based on each statin prescription.

Pairs of *pre-LDL* and *post-LDL* were used to evaluate the effect of statin up-titration, down-titration and non-titration on LDL-C values and LDL-C goal attainment. For effect of statin titration on LDL-C, the mean percentage change and 95% confidence interval (CI) were computed. An analysis to investigate for gender-related differences was also performed.

To investigate the effect of statin titration on LDL-C goal attainment, CVD risk group for each patient was used to set the LDL-C goal (Table 2). These were based on local clinical guidelines [6]. Subsequently, the absolute difference in the LDL-C goal attainment rates were computed before and after titration. For patients in the “Non-titrators” and “No statin” cohorts, absolute difference in the LDL-C goal attainment rates were computed after each statin prescription.

**Table 2:** LDL-C target levels in four risk categories

CVD Risk group based on modified Framingham Risk Calculator	LDL-C goal (mmol/L)
Very high	< 2.1
High	< 2.6
Intermediate	< 3.4
Low	< 4.1

LDL-C target levels based on Ministry of Health Singapore clinical guidelines on lipid disorders. Abbreviations: *CVD* Cardiovascular disease, *LDL-C* Low-density lipoprotein cholesterol.

Multivariate logistic regression was performed to estimate the odds ratio of LDL-C goal attainment for patients in the “Titrators” cohort. The odds ratios were adjusted for various factors such as gender, age and ethnicity. The *P* values were set at *P* < 0.05 for statistical significance. The regression analysis and computation of *p*-values were performed using the Python “statsmodel” package, version 0.12.1.

## Results

**Fig. 2:**
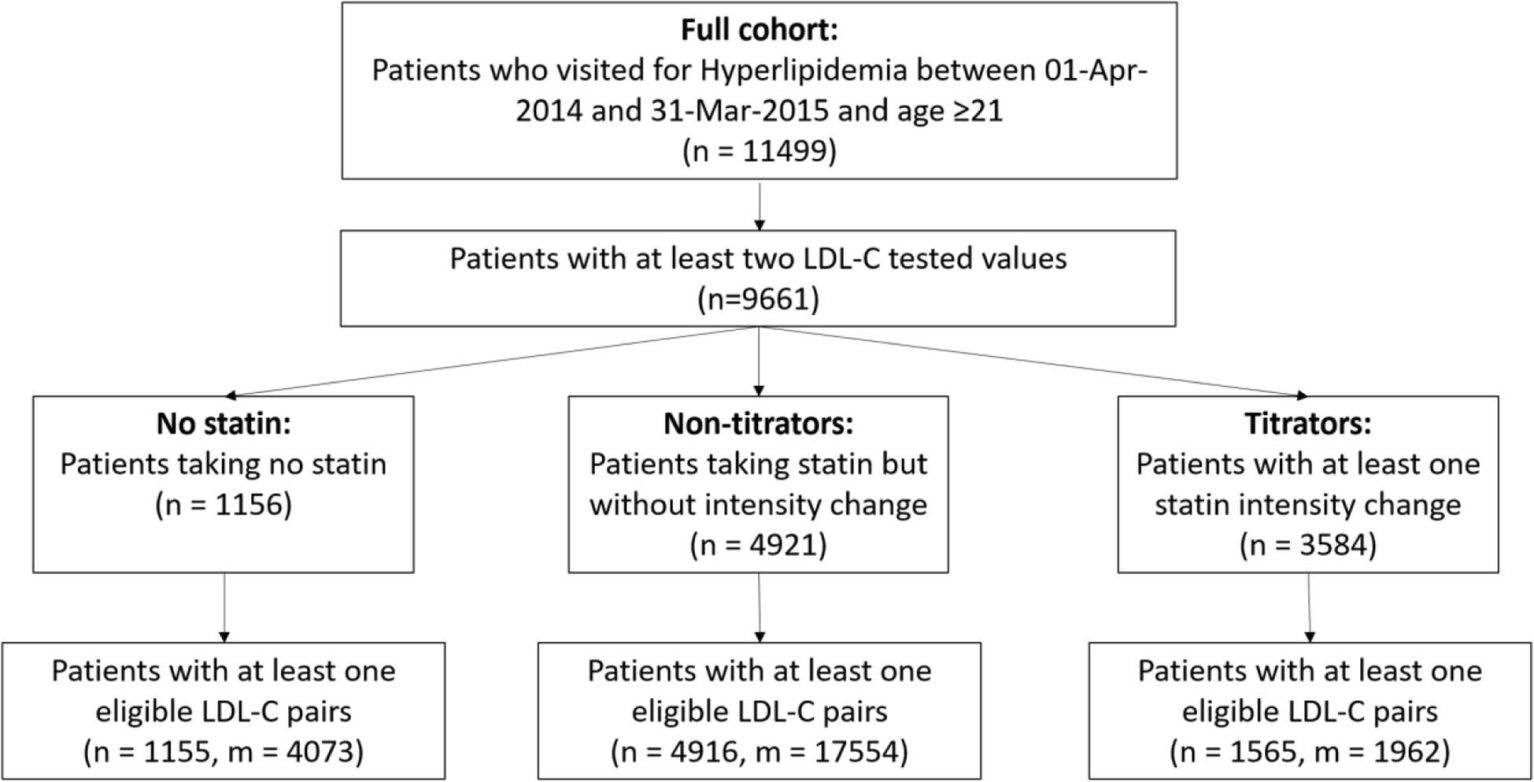
Flow chart illustrating the derivation of the patient cohorts. Abbreviation: *n* number of patients, *m* number of LDL-C pairs

A total of 11,499 unique patients with Dyslipidaemia and 21 years or older were extracted from the polyclinic EMR, consisting a total of 266,762 visits. 9661 of them (84.0%) had at least two LDL-C values over the study period. There were 1155, 4916 and 1561 patients in the “No statin”, “Non-titrators” and “Titrators” cohorts respectively, with at least one LDL-C pair. The number of LDL-C pairs for those in “No statin”, “Non-titrators” and “Titrators” cohorts were 4073, 17,554 and 1962 respectively. Fig. 2 is a flow chart to illustrate the derivation of the patient cohorts.

The baseline characteristics of the patients in each cohort are shown in Table 3. The mean age ranges from 64.8 to 69.0 years across the three cohorts, with slight female predominance (56.8%, 54.5%–60.1%). Most patients (79.1%, 68.7%–84.7%) in each cohort had Hypertension, and more than one-third of patients (36.1%, 13.8%–39.0%) in the “Non-titrators” and “Titrators” cohorts had Diabetes. Around half of the patients (55.0%, 44.7%–62.2%) in each group had Dyslipidaemia for at least 5 years. For patients taking some statin (“Non-titrators” and “Titrators” cohorts), the most prevalent statin intensity at base visit was LI, and most of patients fell into the HR or VHR groups.

**Table 3:** Baseline characteristics of patients in study cohort

Characteristics	No statin (*n* = 1156)	Non-titrators (*n* = 4916)	Titrators (*n* = 1565)	Full cohort (*n* = 11,499)
Total Patients, n (%)	1155 (100)	4916 (100)	1565 (100)	11,499 (100)
Age (year), mean (SD)	65.3 (11.0)	69.0 (10.3)	64.8 (10.0)	67.8 (11.3)
Sex, males, n (%)	461 (39.9)	2008 (40.8)	712 (45.5)	4967 (43.2)
Race, n (%)				
Chinese	1042 (90.2)	4185 (85.1)	1279 (81.7)	9606 (83.5)
Malay	22 (1.9)	282 (5.7)	109 (7.0)	726 (6.3)
Indian	55 (4.8)	236 (4.8)	105 (6.7)	655 (5.7)
Others	36 (3.1)	213 (4.3)	72 (4.6)	512 (4.5)
Diagnosis, n (%)				
Dyslipidaemia	1155 (100.0)	4916 (100.0)	1565 (100.0)	11,499 (100.0)
Diabetes	159 (13.8)	1915 (39.0)	594 (38.0)	4153 (36.1)
Hypertension	793 (68.7)	4166 (84.7)	1120 (71.6)	9101 (79.1)
Years with Dyslipidaemia at base visit, n (%)				
0	196 (17.0)	226 (4.6)	177 (11.3)	1074 (9.3)
1	76 (6.6)	298 (6.1)	125 (8.0)	797 (6.9)
2	81 (7.0)	600 (12.2)	184 (11.8)	1350 (11.7)
3	103 (8.9)	316 (6.4)	121 (7.7)	825 (7.2)
4	183 (15.8)	417 (8.5)	159 (10.2)	1133 (9.9)
> =5	516 (44.7)	3059 (62.2)	799 (51.0)	6320 (55.0)
Statin intensity at base visit, n (%)				
No	1155 (100.0)	0.0 (0.0)	333 (21.3)	2327 (20.2)
Low	0.0 (0.0)	3998 (81.3)	850 (54.3)	7076 (61.5)
Intermediate	0.0 (0.0)	812 (16.5)	349 (22.3)	1867 (16.2)
High	0.0 (0.0)	106 (2.2)	33 (2.1)	229 (2.0)
Patients in each risk group, n (%)				
Low	515 (44.6)	1111 (22.6)	453 (28.9)	2970 (25.8)
Intermediate	196 (17.0)	504 (10.3)	150 (9.6)	1162 (10.1)
High	331 (28.7)	1827 (37.2)	484 (30.9)	3942 (34.3)
Very high	113 (9.8)	1474 (30.0)	478 (30.5)	3425 (29.8)
Number of LDL tests per year, mean (SD)	0.8 (0.3)	0.8 (0.2)	1.0 (0.3)	0.7 (0.3)
Number of statinprescriptions per year, mean (SD)	0.0 (0.0)	2.6 (0.9)	2.4 (1.2)	2.3 (1.2)

Abbreviations: *LDL* Low-density lipoprotein, *SD* Standard deviation

### Effectiveness of statin intensity titration on LDL-C values

The percentage change of LDL-C for the various intensity titrations are shown on Table 4. Initiation of LI, MI and HI statin resulted in a lowering of LDL-C by 21.6% (18.9% to 24.3%), 28.9% (25.0% to 32.7%) and 25.2% (12.8% to 37.7%) respectively. Among the instances of statin initiation, most instances were started on LI statin (m = 311), followed by MI (m = 189) and HI (m = 3344). Up-titration of statin from LI to MI (m = 637) and HI (m = 49) resulted in a lowering of LDL-C by 16.2% (14.3% to 18.1%) and 24.6% (17.8% to 31.5%) respectively. MI to HI up-titration (m = 281,387) resulted in a lowering of LDL-C by 12.4% (9.1% to 15.7%).

**Table 4:** Percentage change of LDL-C with titrations in statin intensity

From	To	LDL-C change (%)	95% CI	m
**No statin**				
No Statin	No Statin	−1.1	(− 1.6, − 0.6)	4073
**Non Titrator**				
Low-intensity	Low-intensity	0.4	(0.0, 0.8)	14,169
Moderate-intensity	Moderate-intensity	0.3	(−0.6, 1.2)	2961
High-Intensity	High-Intensity	0.9	(−2.6, 4.3)	424
**Titrators (Up)**				
No Statin	Low-intensity	−21.6	(−24.3, −18.9)	311
No Statin	Moderate-intensity	−28.9	(−32.7, − 25.0)	189
No Statin	High-Intensity	−25.2	(−37.7, −12.8)	33
Low-intensity	Moderate-intensity	−16.2	(−18.1, − 14.3)	637
Low-intensity	High-Intensity	−24.6	(−31.5, −17.8)	49
Moderate-intensity	High-Intensity	−12.4	(−15.7, −9.1)	281
**Titrators (Down)**				
Low-intensity	No Statin	18.1	(10.0, 26.1)	64
Moderate-intensity	No Statin	32.1	(4.5, 59.7)	22
Moderate-intensity	Low-intensity	13.2	(7.6, 18.9)	261
High-Intensity	No Statin	89.5	(− 125.2, 304.3)	3
High-Intensity	Low-intensity	24.6	(−20.2, 69.3)	10
High-Intensity	Moderate-intensity	18.4	(8.5, 28.2)	102

Abbreviations: *LDL-C* Low-density lipoprotein cholesterol, *m* number of LDL-C pairs

Down-titration of statin from MI to LI (m = 261) resulted in an LDL-C increase of 13.2% (7.6% to 18.9%), while HI to MI (m = 102) resulted in an increase of 18.4% (8.5% to 28.2%). Discontinuation of LI statin (m = 64) and MI statin (m = 22) resulted in an LDL-C increase of 18.1% (10.0% to 26.1%) and 32.1% (4.5% to 59.7%) respectively.

For comparison, among the LDL pairs from patients in the “No statin” and “Non-titrators” cohort, there was no significant LDL-C change. Among the LDL pairs from patients in the “no statin” cohort, the change in LDL-C was − 1.1% (− 0.6%to − 1.6%).

An analysis to investigate for differences in LDL-C lowering between males and females did not show any statistical differences (Supplemental file – Table 1).

### Effectiveness of statin intensity titration on LDL-C goal attainment rate

The change in LDL-C goal attainment for the various intensity titrations are shown in Table 5. Across all types of statin up-titration, 30.5% of LDL values met the LDL-C goal before titration, while 65.5% met the LDL-C goal after titration, representing an increase of 35.3%. The largest increase of 47.1% was achieved when patients who were not on statin were initiated on a MI statin. This was followed by 40.5% improvement for patients who were initiated on a LI statin. Conversely across all types of statin down-titration, 68.8% of the pre-LDL values met the LDL-C goals, while only post-LDL values 59.3% met the LDL-C treatment goals, representing a drop of 9.5%.

**Table 5:** Change in LDL-C goal attainment with titration in statin intensity

From	To	Change in goal attainment (%)	Goal attainment before titration (%)	Goal attainment after titration (%)	m
**No statin**					
No Statin	No Statin	3.3	63.0	66.3	4073
Total for No statin		3.3	63.0	66.3	4073
**Non Titrator**					
Low-intensity	Low-intensity	2.2	77.9	80.1	14,169
Moderate-intensity	Moderate-intensity	3.7	65.1	68.8	2961
High-Intensity	High-Intensity	5.4	48.1	53.5	424
Total for Non-titrator		2.5	75.0	77.5	17,554
**Titrators (Up)**					
No Statin	Low-intensity	40.5	34.4	74.9	311
No Statin	Moderate-intensity	47.1	26.5	73.5	189
No Statin	High-Intensity	39.4	42.4	81.8	33
Low-intensity	Moderate-intensity	33.1	30.5	63.6	637
Low-intensity	High-Intensity	26.5	61.2	87.8	49
Moderate-intensity	High-Intensity	27.4	20.6	48.0	281
Total for Titrators (Up)		35.3	30.2	65.5	1500
**Titrators (Down)**					
Low-intensity	No Statin	−23.4	65.6	42.2	64
Moderate-intensity	No Statin	−13.6	59.1	45.5	22
Moderate-intensity	Low-intensity	−4.2	73.6	69.3	261
High-Intensity	No Statin	−66.7	100.0	33.3	3
High-Intensity	Low-intensity	−20.0	50.0	30.0	10
High-Intensity	Moderate-intensity	−10.8	61.8	51.0	102
Total for Titrators (Down)		−9.5	68.8	59.3	462

Abbreviations: *m* number of LDL-C pairs

Among all types of statin non-titration, the goal attainment rate of pre-LDL and post-LDL values are 75.0% and 77.5%, respectively. Patients in the “Non-titrators” cohort who were on a LI statin had the highest percentage of pre-LDL and post-LDL values that met the target LDL-C values (77.9% and 80.1% respectively). In contrast, patients in the “Non-titrators” cohort who were on HI statin had the lowest percentage of pre-LDL and post-LDL values that met the LDL-C goals (48.1% and 53.5% respectively).

### Odds ratios of LDL-C goal attainment for statin intensity initiation and titration

Table 6 shows the adjusted odds ratio of statin intensity titration compared to no titration in statin intensity for attainment of target LDL-C based on the post-LDL values. Initiation and up-titration of statin intensity had an odds ratio larger than 1 in all permutations. This indicates a strong association between the statin intensity titration and goal attainment. The largest odds ratio of 23.3 was achieved when patients who were not on statin were initiated on a HI statin.

**Table 6:** Adjusted odds ratios of statin intensity up-titration for LDL-C goal attainment

Therapy	Before	After	Odds ratio	95% CI	***P***-value
Up-titration	No statin	Low-intensity	7.1	(4.8, 10.4)	< 0.001
	No statin	Moderate-intensity	13.4	(8.1, 22.0)	< 0.001
	No statin	High-intensity	23.3	(7.1, 76.1)	< 0.001
	Low-intensity	Moderate-intensity	1.9	(1.5, 2.3)	< 0.001
	Low-intensity	High-intensity	6.1	(2.3, 16.6)	0.001
	Moderate-intensity	High-intensity	1.3	(1.0, 1.8)	0.09

Odds ratios adjusted for gender, age, race, CVD risk group, goal attainment of pre_LDL, Diabetes and Hypertension. Abbreviation: *pre_LDL* LDL-C before statin intensity titration

## Discussion

In this study, the LDL-C lowering effect when initiating LI, MI and HI statin was less compared to results from clinical trials [10–15, 27, 28]. The lowering effect was 21.6%, 28.9% and 25.2% respectively for LI, MI and HI statin respectively. In contrast, a systemic review published by the US Agency for Healthcare Research and Quality had quoted a < 30%, 30% to 50% and > 50% reduction across the three intensity bands [3]. One possible reason for this discrepancy is due to suboptimal medication adherence among patients in the real-world [29]. This is supported by findings from another real-world study on LDL-lowering effect by Koren et al., which saw a 35.3% LDL-C reduction on starting high-intensity atorvastatin [30].

Contrary to trial data on incremental statin dose-related LDL-C lowering effect [31], the results reveal that LDL-C reduction is lower when HI statin is initiated compared to the commencement of LI and MI statin therapy. Patients who are started on HI statins are postulated to have lower adherence to their prescription, given the fear of adverse effects with a higher doses [32]. Studies by Grover et al. and Virani et al. have both reported lower adherence of patients to high-intensity statin therapy by 0.4% and 1.9% respectively, compared to those on moderate-intensity [33, 34]. Based on this finding, prescribers may wish to consider selectively initiating statin-naïve patients on LI statins to address their fears and concerns, or alternative lipid lowering therapy such as PCSK9 inhibitors which have been demonstrated to have a positive impact on patients’ adherence as well as quality of life [35, 36].

By performing a weighted average of statin up-titration, our analysis revealed 15.5% reduction in LDL-C for those who had up-titration of statin intensity. This is higher than the 9.6% reduction reported by Toth et al. [18]. We believe this could be explained by genetic variations between Caucasian and Asian populations, resulting in differences in pharmacokinetic and pharmacodynamics effects [37]. A large scale trial has demonstrated that a 5-mg dose of simvastatin to be as effective as the 20-mg dose used in Western countries [38]. A pharmacokinetics study on Rosuvastatin conducted in a Singapore population also found a higher (1.63 to 2.31) area under the plasma concentration-time curve in Asians compared to white subjects, showing higher effects in the former [39].

The results show the efficacy of up-titration in lowering the LDL-C value compared to no titration. There was at least 12.4% LDL reduction for statin up-titration compared to a relatively modest 1.1% reduction for no titration. This is to be expected as constant dose of statin is typically used to maintain LDL-C rather than lower it.

In addition to LDL-C lowering, odds ratio provides information to facilitate the decision-making process on statin titration from the LDL-C goal attainment perspective. When compared to the findings by Toth et al. which reported odds ratios of statin up-titration on LDL-C goal attainment ranging from 1.8 to 2.9, this study showed similar results (OR = 1.3 to 6.1). Toth et al. had focused on patients with high risk CVD and also used a more stringent LDL-C target of < 1.8 mmol/L for some patients compared to this study [18].

### Study strength and limitations

Analysis of real-world data of a captive population of patients constitutes a strength in this study. This allows us to account for the effects of real-world practicalities such as correlations between potentially suboptimal medication adherence and effectiveness of statin intensity adjustment. This real-world evidence could be used to complement results from clinical trials in setting more realistic expectations for both clinicians and patients on the attainment of LDL-C treatment goals.

Another benefit of using real-world data is the opportunity to gain insight on the impact of down-titrating and discontinuing statins on LDL-C levels. Such changes would be challenging to elucidate from clinical trials due to obvious ethical reasons. These practices may have happened based on physicians’ recommendation when patients encounter adverse effects from the medication, or by patients’ own volition without physician recommendation. Such behaviour was common, resulting in 376 instances of statin down-titration and 86 instances of statin discontinuation. These results provide an estimation of potential rise in LDL-C level when patients discuss the option of reducing or discontinuing their statin therapy. In contrast to elevation in statin intensity, the magnitude on LDL-C increase was lower for down-titration and discontinuation than up-titrating and initiation. This may be an avenue for further research into a possible sustained effect of statin on LDL-C even after dose-reduction or discontinuation.

This study also has its limitations. Firstly, in concluding that the LDL-C lowering effect was lesser in than found in trial-based studies, we observe that these trial-based studies have mostly been on Caucasian populations. Therefore our conclusion may not be applicable to non-Asians considering the differences in genetic make-up on statin pharmacology. Secondly, only data from a single site was used in the analyses. This study could follow-up with an analysis across multiple study sites in future, which would provide larger cohort size for more in-depth subgroup analyses.

Looking ahead, the results from this study provide reference for a patient decision support tool to be developed to help facilitate shared decision-making in statin therapy in Asians. This tool is envisioned to assist patients and clinicians when selecting, initiating and adjusting statin therapy, so as to achieve best possible clinical outcomes and minimal adverse effects.

## Conclusions

This study provides real-world evidence to elucidate the effect of statin intensity titration on LDL-C levels in patients with Dyslipidaemia. The real-world data in primary care showed lower LDL-C reduction after raising the statin intensity compared to results reported in clinical trials. In addition, this study also provided insights into statin down-titration and discontinuation, which had lower impact on LDL-C level and LDL-C goal attainment than its corresponding up-titration or initiation. In clinical practice, these findings should be taken into consideration and provide further insight to clinicians when making statin adjustment recommendations in order to achieve LDL-C targets.

## Supplementary information

**Additional file 1: Table S1.** Percentage change of LDL-C with titrations in statin intensity (by gender).

## Data Availability

The datasets analysed during the current study are not publicly available as they contain information that are sensitive to the study institution. They may be made available from the corresponding author on reasonable request.

## Abbreviations

ACC: American College of Cardiology
AHA: American Heart Association
CVD: Cardiovascular diseases
EAS: European Atherosclerosis Society
EMR: Electronic medical records
ESC: European Society of Cardiology
HI: High-intensity
HMG-CoA: Hydroxyl-3-methylglutaryl coenzyme A
HR: High cardiovascular risk
ICD-10: International Classification of Diseases, 10th Revision
LDL-C: Low-Density Lipoprotein-Cholesterol
LI: Low-intensity
LR: Low cardiovascular risk
MI: Moderate-intensity
MR: Medium cardiovascular risk
VHR: Very high cardiovascular risk
