# Supplementary material for: LDL-cholesterol change and goal attainment following statin intensity titration among Asians in primary care: a retrospective cohort study
Source: Lipids Health Dis. 2021 Jan 6;20:2. doi: 10.1186/s12944-020-01427-z (PMC7788928; doi:10.1186/s12944-020-01427-z)
Supplement: Supplementary file 1 — Additional file 1: Table S1. Percentage change of LDL-C with titrations in statin intensity (by gender). [file 12944_2020_1427_MOESM1_ESM.docx]

**Supplemental file -** Table S1. Percentage change of LDL-C with titrations in statin intensity (by gender).

| From | To | | LDL-C change for males(%,m) | LDL-C change for females(%,m) | p |
| --- | --- | --- | --- | --- | --- |
| **No statin** | | | | | |
| No Statin | No Statin | -1.0 (1651) | | -1.2 (2422) | 0.7561 |
| **Non Titrator** |  | |  |  |  |
| Low-intensity | Low-intensity | | 0.3 (5416) | 0.5 (8753) | 0.6220 |
| Moderate-intensity | Moderate-intensity | | -0.4 (1381) | 0.9 (1580) | 0.1559 |
| High-Intensity | High-Intensity | | 0.4 (241) | 1.5 (183) | 0.7710 |
| **Titrators (Up)** | | | | | |
| No Statin | Low-intensity | | -20.2 (105) | -22.3 (206) | 0.4737 |
| No Statin | Moderate-intensity | | -28.7 (81) | -29.0 (108) | 0.9312 |
| No Statin | High-Intensity | | -26.6 (18) | -23.6 (15) | 0.8155 |
| Low-intensity | Moderate-intensity | | -15.9 (295) | -16.4 (342) | 0.7993 |
| Low-intensity | High-Intensity | | -26.1 (31) | -22.0 (18) | 0.5445 |
| Moderate-intensity | High-Intensity | | -14.0 (152) | -10.5 (129) | 0.2963 |
| **Titrators  (Down)** | | | | | |
| Low-intensity | No Statin | | 15.4 (28) | 20.1 (36) | 0.5514 |
| Moderate-intensity | No Statin | | 40.7 (9) | 26.2 (13) | 0.6431 |
| Moderate-intensity | Low-intensity | | 10.1 (100) | 15.2 (161) | 0.3371 |
| High-Intensity | No Statin | | 137.5 (2) | -6.4 (1) | NA |
| High-Intensity | Low-intensity | | 46.7 (4) | 9.8 (6) | 0.4694 |
| High-Intensity | Moderate-intensity | | 19.8 (48) | 17.1 (54) | 0.7857 |

Abbreviations: LDL-C = low-density lipoprotein cholesterol, m = number of LDL-C pairs, NA = not applicable.
